# COVID‐19 infection in CVID patients: What we know so far

**DOI:** 10.1002/iid3.450

**Published:** 2021-05-12

**Authors:** Niels Weifenbach, Alisha Jung, Stefan Lötters

**Affiliations:** ^1^ Trier University, FB VI, Biogeography Trier Germany; ^2^ Department of Medical Science Private Universität im Fürstentum Liechtenstein Triesen Liechtenstein

**Keywords:** common variable immunodeficiency, convalescent plasma, immunoglobulin replacement, SARS‐CoV‐2

## Abstract

**Introduction:**

In patients with common variable immunodeficiency (CVID), immunological response is compromised. Knowledge about COVID‐19 in CVID patients is sparse. We, here, synthesize current research addressing the level of threat COVID‐19 poses to CVID patients and the best‐known treatments.

**Method:**

Review of 14 publications.

**Results:**

The number of CVID patients with moderate to severe (~29%) and critical infection courses (~10%), and the number of fatal cases (~13%), are increased compared to the general picture of COVID‐19 infection. However, this might be an overestimate. Systematic cohort‐wide studies are lacking, and asymptomatic or mild cases among CVID patients occur that can easily remain unnoticed. Regular immunoglobulin replacement therapy was administered in almost all patients, potentially explaining why the numbers of critical and fatal cases were not higher. In addition, the application of convalescent plasma was demonstrated to have positive effects.

**Conclusions:**

COVID‐19 poses an elevated threat to CVID patients. However, only systematic studies can provide robust information on the extent of this threat. Regular immunoglobulin replacement therapy is beneficial to combat COVID‐19 in CVID patients, and best treatment after infection includes the use of convalescent plasma in addition to common medication.

Common variable immunodeficiency (CVID) is a primary immunodeficiency in humans. Typically, the immunological response is disrupted due to hypogammaglobulinemia and B and T cell abnormalities. Patients commonly suffer from a wide array of additional clinical features. Common health issues include recurrent airway infections, autoinflammatory or neoplastic disorders, and autoimmune problems.^1^, ^2^ CVID patients under regular immunoglobulin (IG) replacement often remain stable.^2^, ^3^, ^4^

CVID is considered the most widespread of all primary immunodeficiencies, and virus infections can be perilous to patients.^2^, ^4^, ^5^ Therefore, in light of the ongoing coronavirus disease 2019 (COVID‐19) pandemic, it is of particular interest to understand how CVID patients respond to infection with its agent, SARS‐CoV‐2. Though many people infected with the virus are asymptomatic or develop only mild to moderate symptoms, a fraction of patients develops severe to critical symptoms (e.g., drastic hypoxemia and acute respiratory distress syndrome), which are fatal in some cases.^5^, ^6^, ^7^ So far, several cases of COVID‐19 in CVID patients have been reported, with both nonserious and serious infection course. However, many of these accounts refer to single case reports of hospitalized patients,^3^, ^8^ whereas systematic cohort‐wide data are sparse.^5^, ^7^ The purpose of this paper is to sort the published information available to date and to contribute to answering the questions: (i) How threatening is COVID‐19 to CVID patients? and (ii) what is the most effective treatment?

On the basis of a search in Medline, EMBASE, PubMed, DIMDI, Google Scholar, and Web of Science (February 2021) for “CVID,” “COVID” (and; 2020–2021), we found 14 publications describing COVID‐19 infections in CVID patients (Table S1). The total number of such cases was 68 (in all cases, SARS‐CoV‐2 was detected via PCR test from swabs), aged 22–68 (mean, 47.3), almost equally distributed among males and females. Most of them had comorbidities (*n* = 53) related to CVID (involving the upper or lower respiratory tract in 33 individuals), or even other health problems, and were usually under IG replacement therapy (*n* = 48). We divide COVID‐19 etiopathology into three categories: (i) Mild infection (patient not necessarily hospitalized, if so, no oxygen supply necessary) was noticed in 39 patients. We allocate also two patients to this group that were asymptomatic (ages 54 and 56). (ii) Moderate to severe infection (hospitalization, oxygen supply necessary but no intubation) was recorded in 20 patients. (iii) Critical infection (hospitalization, intubation necessary) was observed in seven patients. Apparently, there was no effect of age as the youngest patients with a critical infection were 37‐ and 42‐years old, respectively. In total, nine patients died (between 35‐ and >75‐years old): One in the category of mild, five in that of moderate, and three in that of critical infection. In the remaining, recovery time greatly varied (5–33 days) and several patients remained SARS‐CoV‐2 positive after recovery (at maximum for >2 months), while free of symptoms.

COVID‐19 symptoms typically included fever (*n* = 52), cough (*n* = 49), and shortness of breath/dyspnea (*n* = 27) among others (e.g., myalgia, fatigue, and intestinal problems). No treatment was given in 17 patients with asymptomatic courses or mild infection, whereas antibiotics were provided in 40 and chloroquine/hydroxychloroquine in 32 cases. Antiviral treatment (e.g., lopinavir, retinavir, and remdisivir) was administered in 14 patients. IG was administered in seven, monoclonal antibodies (e.g., tocilizumab) in eight, and convalescent plasma in eight patients.

Among CVID patients, the proportions of both moderately to severely (~29%) and critically infected patients (~10%), and those who died from COVID‐19‐related complications (~13%), are elevated compared to general infection‐fatality rates among human populations.^9^ However, these comparisons might result in overestimates. Due to the limited availability of systematic cohort assessments, asymptomatic or mild COVID‐19 infections in CVID patients—that obviously occur—are mostly ignored.^5^, ^7^ It has been suggested that the incidence of such cases may even be increased among CVID patients because of deficient B lymphocyte response and loss of interleukin‐6.^4^ Moreover, especially in the future, CVID patients under regular IG therapy may be provided with antibodies before infection, preventing a critical infection. Perhaps rather than SARS‐CoV‐2 itself and the subsequent immune reaction of the body,^6^ secondary bacterial infections may be the more concerning issue in CVID patients. This could potentially explain why two‐thirds of the fatal cases reported had moderate to severe infection courses only (Table S1). For these reasons, regular IG replacement and early hospitalization during COVID‐19 infection might increase survival in CVID patients,^3^ along with common treatments (e.g., antiviral therapy).^6^ In addition, the application of convalescent plasma was demonstrated to have positive effects.^10^ Only two patients receiving it died; one was off IG before infection, whereas the other suffered from lymphoma and cancer.

## CONFLICT OF INTERESTS

The authors declare that there are no conflict of interests.

## AUTHOR CONTRIBUTIONS

Niels Weifenbach and Stefan Lötters conceptualized and designed the study. Niels Weifenbach and Alisha Jung contributed to the data acquisition. All authors contributed to the analysis and interpretation of the data and wrote the final manuscript. Stefan Lötters supervised the work.

## Supporting information

Supporting information.

## Data Availability

The data that supports the findings of this study are available in the supplementary material of this article (Table S1).
